# Lectures for Mothers

**Published:** 1921-05-28

**Authors:** 


### Lectures for Mothers.

A few days ago a London coroner held an inquest on a child of twelve months who had died from convulsions, said to have been brought about by malnutrition. "I don't know," commented the coroner, "whether there is a doctor in this district who would feel inclined to deliver a course of lectures to mothers on the feeding of the children. It seems to be an urgent necessity to have such lectures." He added, in the tone of one dismissing a painful subject from his mind, "But there! I don't suppose mothers would attend even if such a course were announced." We quote these remarks, as they were published in several newspapers, because they have a direct bearing on two articles which recently appeared in these columns. In the first, several weeks ago, we suggested that general practitioners might do a great deal of valuable preventive work on their own initiative by giving periodical lectures to their patients—for instance, at the very beginning of an outbreak of local epidemic disease. In the second we discussed various ways in which the Ministry of Health and local health authorities might conduct a publicity campaign in order to emphasise the importance of systematically combating disease and to make clear the principles to be adopted in order to keep healthy. We are convinced that the Ministry of Health, whose enlightened enterprise in many directions we have never been slow to recognise, has not yet realised the immense opportunities that lie ready at hand for a continuous and progressive system of first-class propagandism. The great health organisation of the country which it controls is an instrument at once available for carrying out such a policy on a bold scale, and there was never a time when the public was more receptive of ideas for keeping healthy by living healthily. The United States have set this country a gallant example of the right methods to employ in keeping the vital question of its physical well-being constantly in the notice of the community, and many of the methods that we have cited in an earlier issue were drawn from American practice. That the unwise and premature assumption of the London coroner as to the lack of response on the part of the public is to some extent shared by the Ministry of Health seems to be proved by Sir Alfred Mond's unwillingness to adopt so simple a device as to issue food pamphlets for the multitude. To adopt this kind of negative argument is to think back a quarter of a century. The experience of hundreds of voluntary health workers—many of whom are personally known to us—conclusively proves the existence of a real and growing eagerness on the part of large numbers of people, not least among working-class mothers, to gain some practical knowledge of the laws of health. The school clinic has done almost as much as anything to break down the barriers of parental ignorance and prejudice; and the welfare work in industry, as well as the infant welfare centres established in all parts of the country, have met with a great deal of success and done an astonishing amount of good that could not have been possible without the co-operation of those on whose behalf the work was started. There is certainly no warrant for the statement that mothers would not attend simple medical lectures designed to help them in the upbringing of their children. They have, in fact, already done so in the most unlikely town areas. All that is required in most cases to get them together for such a purpose is a suitable choice of the day, the time and the place of lecturing, and a little friendly encouragement and advice by health visitors and others. There is ignorance enough, in all conscience (and not only among the poor) about the elements of hygiene; and the dull acquiescence in the dismal consequences of ignorance may momentarily baffle individual workers. But here is a great work for the State to do. And the Department which represents the State will succeed not by shaking its head over the hopelessness of its task, but only by energetic action. Ignorance is ripe for its conquest by knowledge.

